# Lithium Titanate/Carbon Nanotubes Composites Processed by Ultrasound Irradiation as Anodes for Lithium Ion Batteries

**DOI:** 10.1038/s41598-017-06908-3

**Published:** 2017-08-08

**Authors:** João Coelho, Anuj Pokle, Sang-Hoon Park, Niall McEvoy, Nina C. Berner, Georg S. Duesberg, Valeria Nicolosi

**Affiliations:** 1 School of Chemistry/CRANN, Trinity College Dublin, College Green, Dublin 2, Ireland; 2 School of Physics/CRANN, Trinity College Dublin, College Green, Dublin 2, Ireland; 3 Institute of Physics, EIT 2, Faculty of Electrical Engineering and Information Technology, Werner-Heisenberg-Weg 39, 85577 Neubiberg, Germany

## Abstract

In this work, lithium titanate nanoparticles (nLTO)/single wall carbon nanotubes (SWCNT) composite electrodes are prepared by the combination of an ultrasound irradiation and ultrasonic spray deposition methods. It was found that a mass fraction of 15% carbon nanotubes optimizes the electrochemical performance of nLTO electrodes. These present capacities as high as 173, 130, 110 and 70 mAh.g^−1^ at 0.1C, 1C, 10C and 100C, respectively. Moreover, after 1000 cycles at 1C, the nLTO/SWCNT composites present a capacity loss of just 9% and a Coulombic efficiency of 99.8%. Therefore, the presented methodology might be extended to other suitable active materials in order to manufacture binder free electrodes with optimal energy storage capabilities.

## Introduction

Lithium ion batteries (LIB) play a major role in portable technology, energy storage/conversion systems and are currently being proposed for potential applications in electric vehicles^1, 2^. Due to its relatively high capacity, graphite has been widely used as a LIB anode active material. However, graphite experiences a volume expansion of 10% during the charge-discharge process which leads to significant capacity fading^3, 4^. In this context, a zero strain material, such as spinel lithium titanate (LTO), is promising alternative for Li-ion battery negative electrode. The high structural stability of LTO usually assures a good lithium insertion/extraction reversibility, better rate capability, less capacity loss upon cycling and an enhanced performance over time^5–7^. LTO also exhibits a high lithium insertion potential (1.55 V vs. Li^+^/Li) in comparison with conventional graphite electrodes. Therefore, even at low temperatures or high current rates, lithium plating is very unlikely to occur^3, 8^. Thus, regarding safety issues, LTO represents a much better option than the current technology^7, 8^. Besides all of the aforementioned advantages, lithium titanate is also cheap and non toxic, thus it can be used in large scale industrial applications. In the other hand, LTO high insertion potential (and a relatively low theoretical capacity of 175 mAh.g^−1^) leads to a lower cell energy density. Nevertheless, as LTO can be safely used at high current rates, it is still a good candidate for applications where the main concern is high power density rather than energy density^9, 10^.

Currently, most of the work done in LTO based systems focus on developing electrodes configurations that mitigate the detrimental effects of LTO poor Li^+^ diffusion coefficient (10^−6^ cm^2^.s^−1^) and very low electronic conductivity (10^−13^ S.cm^−1^). These shortcomings can be addressed by designing electrode configurations based on nano sized particles and/or systems with high porosity in good contact with conductive carbon materials, such as graphene^8, 11, 12^ and carbon nanotubes^13, 14^. Nanoparticles usually provide shorter diffusion path for both lithium ions and electrons, in comparison with the bulk counterpart, thus compensating for the low lithium diffusion coefficient of LTO^7, 10, 15, 16^. The conductive carbon additives compensate for the low electrical conductivity of LTO, thus enabling high rate performance^7^. For instance, Haetge *et al*.^7^ prepared highly porous films based on Li_4_Ti_5_O_12_ nanoparticles that present a capacity of ~150 mAh.g^−1^ at a current rate as high as 64C. K. Naoi *et al*.^16, 15^ reported that small nano-crystalline particles of LTO synthesized via sol-gel and anchored on carbon fibers can operate at an unusually high current density of 1200C while retaining a relatively high capacity of 80 mAh.g^−1^. However, these synthesis approaches are just feasible at laboratory scale and might not be practical at an industrial level. In this paper we present a simple and cost-effective ultrasound based methodology that can be successfully used for electrodes manufacture. Moreover, this methodology is also highly scalable in comparison to other traditional synthesis methods^17^. In fact, for the last couple of years, ultrasounds have been widely used to prepare highly stable dispersions of two dimensional nanomaterials^18, 19^. Regarding energy storage systems, graphene^20^ and MoO_3_^21^ nanosheets processed by ultrasound irradiation have been successfully tested for thin film supercapacitor applications. The authors have as well recently reported the manufacture of a symmetric supercapacitor based on a MnO_2_/Graphene composite exfoliated in one single step approach^19, 22^. Briefly, this approach relies on the delamination of bulk layered crystals and subsequent stabilization of the obtained two dimensional materials according to solubility parameter laws. Due to the nature of the materials used, this method is usually referred as liquid phase or chemical exfoliation. However, the material structure should not represent a limitation for eventual applications. As LTO is not a layered material, *liquid phase exfoliation* does not seem to be an appropriate terminology for the processing technique. Therefore, in order to avoid ambiguity and perhaps some confusion, the LTO processing technique will be referred as ultrasound treatment/irradiation or liquid phase processing. In this work, LTO (Linyi Gelon LIB Co., Ltd.) was subjected to an ultrasound treatment, similar to the one described in previous papers^18, 23^ followed by LIBs assembly and testing.

## Results

### Lithium Titanate Sonication and Dispersions Characterization

**Figure 1:**
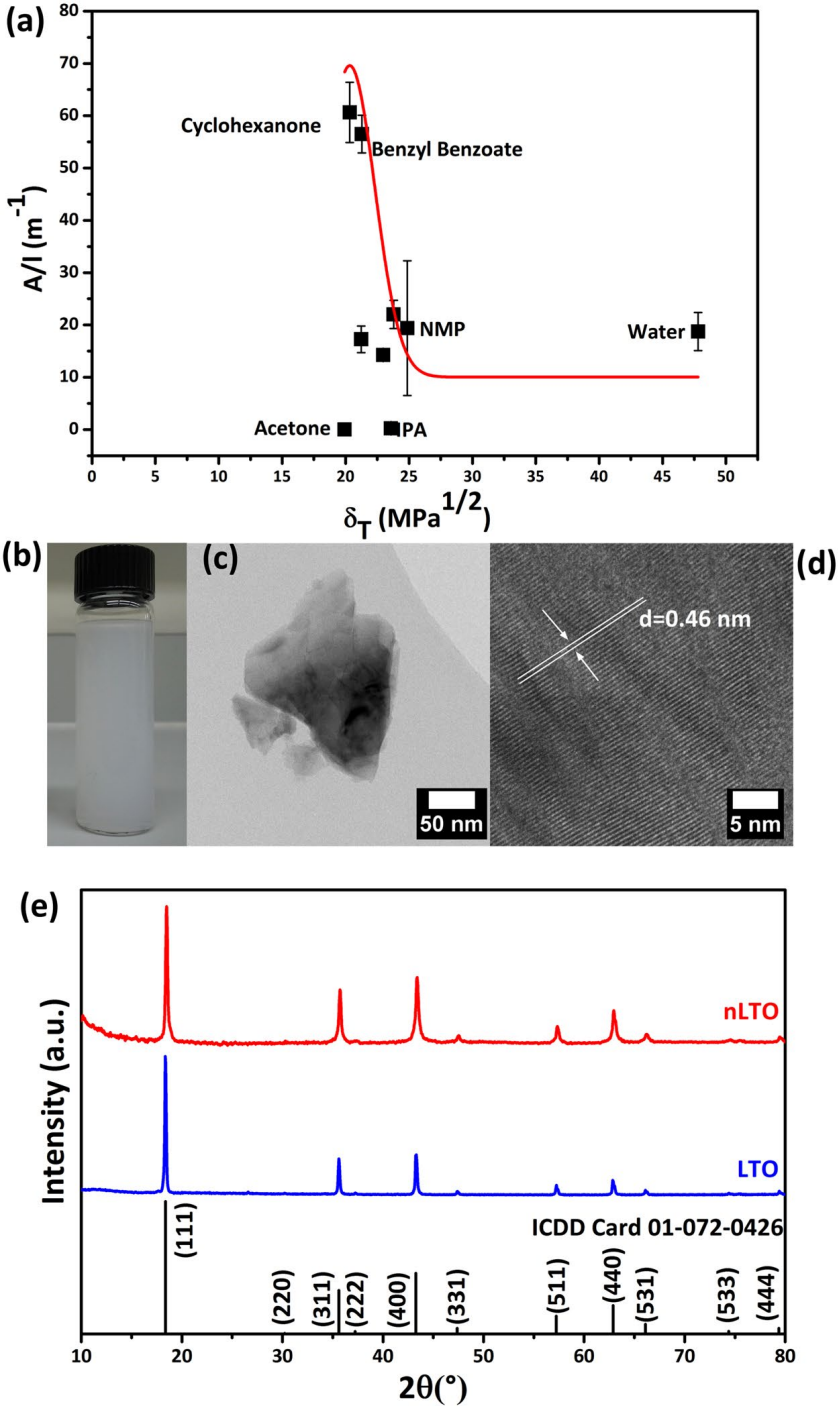
Absorption measurements of nLTO dispersions in a wide range of solvents plotted against solvent Hildebrand solubility parameter, *δ*_*T*_, (**a**); water based nLTO dispersion (**b**); representative TEM micrograph of a nLTO particle (**c**); high resolution TEM of a nLTO particle with corresponding interplanar spacing (**d**) and raw LTO and nLTO XRD pattern (**e**).

The quality of the obtained nLTO dispersions was studied by UV-Vis absorption, as shown in Fig. 1a. The data point distribution presents an “envelope” shape, which is quite similar to other previously reported studies on MoS_2_ and WS_2_ dispersions^18^. In this case it seems that optimal solvents for LTO dispersing should present Hildebrand solubility parameters in the range 15 ${{\rm{MPa}}}^{\frac{1}{2}}$ ≤ δ_*T*_ ≤ 25 ${{\rm{MPa}}}^{\frac{1}{2}}$. From Fig. 1a it is also possible to observe that nLTO could be dispersed in water as well. The obtained dispersions (Fig. 1b) with a concentration as high as 0.1 mg.mL^−1^. are composed of particles with an average size of 174 nm (Fig. 1c). These particles are much smaller than the original LTO, due to the ultrasonication process (see Supplementary Fig. S1). In Fig. 1d is shown a high resolution TEM (HRTEM) micrograph of nLTO. The measured interplanar spacing of 4.6 Å is consistent with other reported values for the (111) plane of spinel LTO^24^. These results were further supported by XRD analysis. The pattern shown in Fig. 1e reveal that the LTO dispersions are composed of a spinel cubic form of Li_0.33_Ti_1.66_O_4_ (ICDD file 01-072-0426) in the space group Fd-3m (227) with a cell parameter of *a* = 8.357 Å. No phase changes were observed after the sonication process, therefore it is possible to conclude that nLTO is a pure phase sample. Further structural characterization of nLTO by Raman and XPS corroborates the XRD results (see Supplementary Figs S2 and S3).

**Figure 2:**
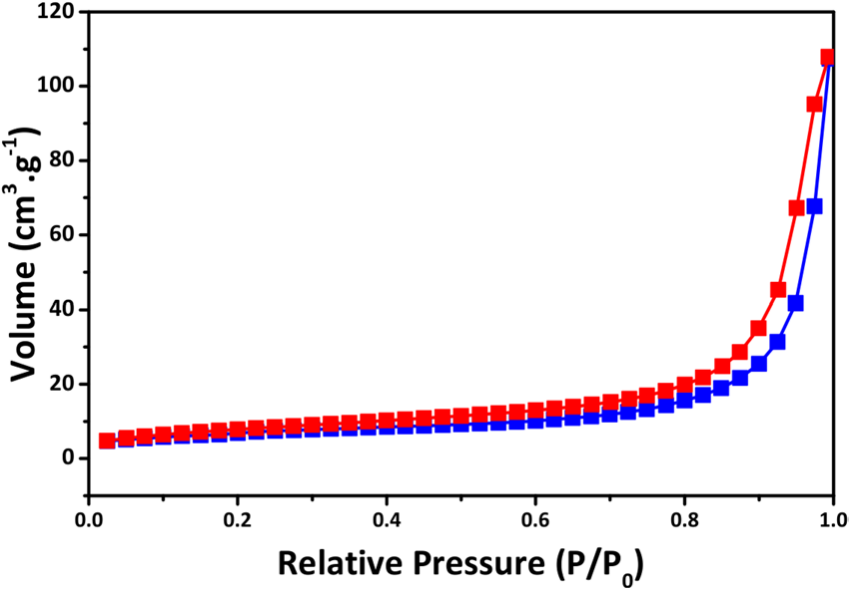
nLTO nitrogen adsorption (red) and desorption (blue) isotherms.

The surface area of nLTO was measured based on multipoint BET method. Analysis of the nitrogen adsorption isotherms (Fig. 2), revealed a surface area of 26.1 m^2^.g^−1^. Surface areas as high as 77 m^2^.g^−1^ were obtained for LTO nanoparticles synthesized via a sol-gel route^25^. LTO nanotubes^26^ and nanowires^27^ with surface areas of 53.69 m^2^.g^−1^ and 38 m^2^.g^−1^, respectively, were also reported. However, all of these nanostructures were prepared by chemical routes that allow fine control of both surface area and particle size in the nano range. Larger particles usually present surface areas in the range of 10 to 25 m^2^.g^−1^ ^1, 28^. This result suggests that nLTO particles are dozens or hundreds of nanometer wide, in good agreement with Fig. 1c.

### nLTO/Carbon Nanotubes Composites Preparation and Characterization

Following the initial morphological and structural characterization, nLTO was tested as a possible active material for LIB anodes. In order to compensate for its intrinsic low electric conductivity (10^−13^ S.cm^−1^), nLTO was mixed with carbon nanotubes (SWCNT) and composite electrodes manufactured by ultrasonic spray deposition. It is important to mention at this point, that for better mixing and deposition, both nLTO and SWCNT were dispersed in 2-Propanol (IPA).

**Figure 3:**
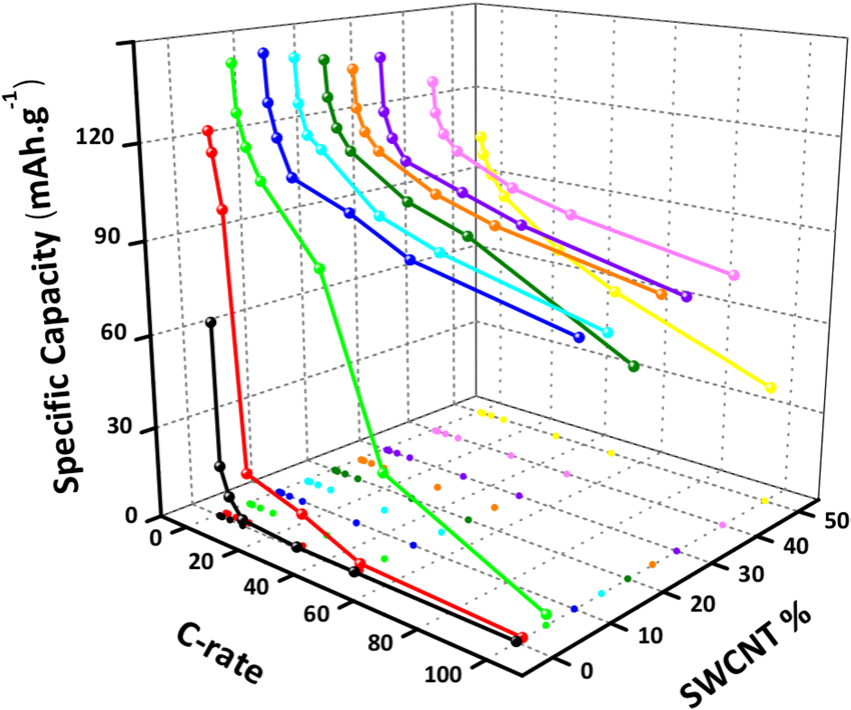
nLTO/SWNCT composites discharge capacities (3^*rd*^ cycle) dependence on the carbon nanotubes mass fraction at several different currents.

Due to their unique physical-chemical properties, SWCNT have been widely used as conductive networks for several electrochemical applications. Carbon nanotubes not only optimize the overall electrical conductivity, but also stabilize the electrode’s active material. This enhancement of mechanical properties is of major importance as it results in an improved electrochemical performance over time, more so if volumes changes occur during the charge/discharge process^29^. According to four point probe measurements, it was found that only a very small amount of carbon nanotubes, ~0.15 wt%, is required to massively improve the electrical conductivity of nLTO/SWCNT composites (see Supplementary Fig. S4). However, this value is only an indication of the SWCNT mass fraction required to establish the first conductive pathways in the sample. In order to optimize the composites electrochemical performance, several electrodes (≈200 *μ*g.cm^−2^) presenting different mass fractions of nanotubes were manufactured and tested by galvanostatic charge/discharge experiments. The results are shown in Fig. 3. It is clear that the gravimetric capacity of nLTO/SWCNT composites strongly depends on the added mass fraction of carbon nanotubes. For bare nLTO electrodes, the capacity at 1C (35 *μ*A.cm^−2^) quickly drops from 64 mAh.g^−1^ to 17.2 mAh.g^−1^ at 2C (70 *μ*A.cm^−2^). This poor electrochemical performance clearly reflects the low conductive properties of nLTO. Upon the addition of 1% wt of nanotubes, the capacity doubles at 1C (34 *μ*A.cm^−2^), and the electrode still maintains a relatively high capacity at 5C (167 *μ*A.cm^−2^), dropping down to zero only at 50C (1.7 mA.cm^−2^). The capacity enhancement at such low mass fractions of nanotubes is in good agreement with the performed percolation studies (see Supplementary Fig. S4). Above a mass fraction of 0.15%, more conductive networks are established, resulting in a better rate capability. As shown in Fig. 3 a maximum capacity of (144 mAh.g^−1^ at 1C (34.2 *μ*A.cm^−2^) is achieved for a CNT mass fraction of 15%. At this stage, a wider conductive network can actually connect more particles in the system, enabling more efficient transport of stored charge to and from the current collector and therefore the capacity is maximized. Higher nanotube contents no longer improve the composite capacity, in contradiction to what might have been expected^30^.

It is likely that above the optimal mass fraction point, the capacity cannot be further optimized as the nLTO maximum electrochemical utilization was already achieved and SWCNT present a much lower specific capacity (see Supplementary Fig. S5). Thus, further addition of nanotubes to the composite, results in the loss of gravimetric capacity. A similar behavior was observed by Higgins *et al*.^30^ for MnO_2_ nanosheets/SWCNT composites. However, the specific capacity evolution as function of SWCNT mass fraction does not follow a clear and very well defined trend. In fact, non-negligible experimental error might cast some doubt on the interpretation of the obtained results (see Supplementary Fig. S6). Nevertheless, the observed optimal mass fraction of ~15% is in good agreement with other literature reports, that mention optimal mass fractions of conductive additive in the range 5–25% wt.^27, 31, 32^.

### Electrochemical Performance of nLTO/SWCNT Composites

**Figure 4:**
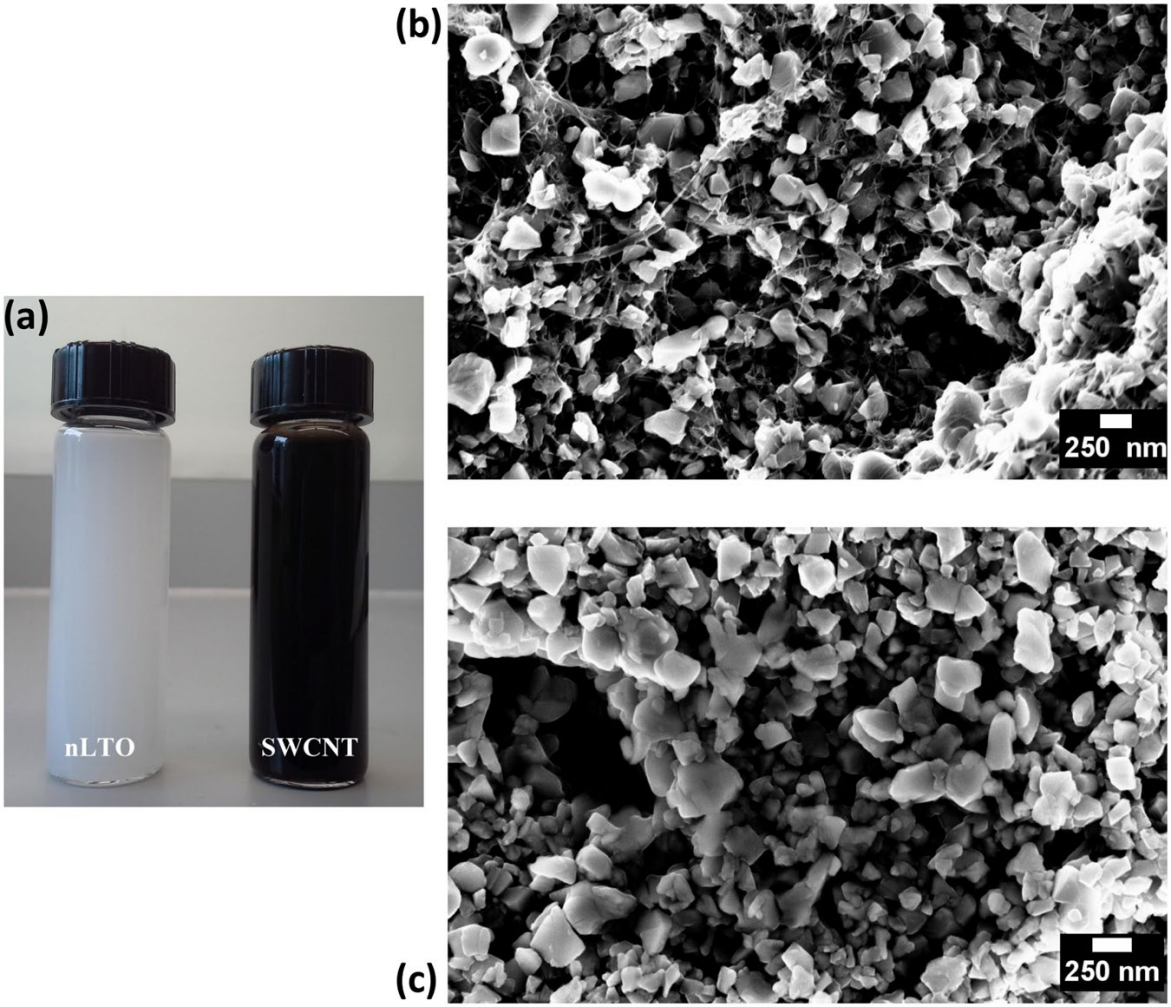
Samples of processed LTO and debundled carbon nanotubes (concentrations ~0.1 mg.mL^−1^) (**a**) and representative SEM micrograph of nLTO/SWCNT (**b**) and nLTO (**c**) sprayed electrodes.

Nanoparticular dispersions in IPA (Fig. 4a) were used to prepare optimized composite electrodes (nLTO:SWCNT (85:15 wt%)). The SEM micrographs shown in Fig. 4b reveal that nLTO/SWCNT electrodes are porous structures composed of lithium titanate particles entangled in a mesh of SWCNT. The presence of a carbon nanotube network is highlighted when the composites and bare electrode’s micrographs (Fig. 4c) are compared. The porosity of the composites is of major importance as it allows a better diffusion of the electrolyte through the electrode^7^. Moreover, the nLTO particles do not seem to aggregate as much as other 2-D materials, resulting in a better electrochemical utilization of the active material.

**Figure 5:**
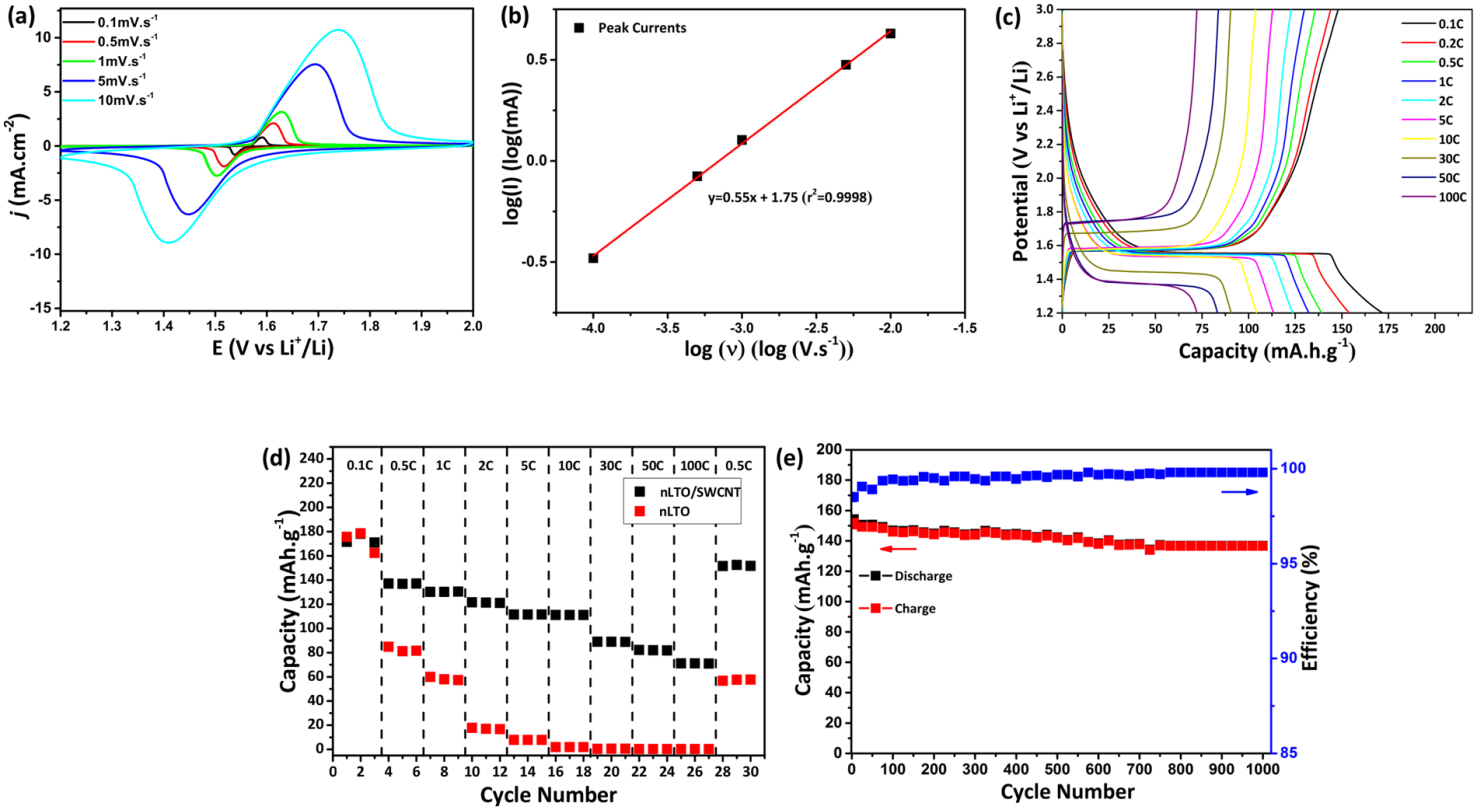
Electrochemical properties of nLTO/SWCNT composites: cyclic voltammograms acquired between 1.2 V and 2 V at different scan rates (**a**); linear fitting for the CV peaks intensity as function of the applied scan rate (**b**); representative charge-discharge curves at several currents (**c**); rate capability (**d**) and cycling performance cycling performance for 1000 cycles at 1C (3.30 *μ*A.cm^−2^) (**e**).

The electrochemical performance of the optimized composites was studied by cyclic voltammetry and galvanostatic charge-discharge cycling in a 1 M LiPF_6_ ((EC:DMC)(1:1)) electrolyte against lithium metal. In Fig. 5a are presented the composites cyclic voltammograms (CVs) acquired at several scan rates in the range 1.2 V to 2 V. The characteristic cathodic and anodic peaks associated with the Li^+^ insertion/extraction in spinel Li_4_Ti_5_O_12_^5, 7, 24, 33^. (Ti^4+^/Ti^3+^ redox couple) can clearly be seen and are still well defined even at scan rates of 10 mV.s^−1^. In fact, the shape of these peaks is widely used to characterize the kinetics of the electrochemical processes. For Li^+^ insertion/extraction, sharp and well defined peaks are associated with fast reaction kinetics, while slow or poor electrochemical processes give rise to broad peaks^34^. For the composites under study, the shape of these CVs is quite stable upon increasing scan rate, thus suggesting good Li^+^ insertion/extraction kinetics in nLTO. However, a sloping of the anodic peaks at higher scan rates is observed, suggesting a sluggish electrochemical process for higher scan rates^9^. Cyclic voltammetry can also be used to test the reversibility of an electrochemical system. An ideal reversible system requires that the ratio between the anodic and cathodic peaks intensity $\frac{{I}_{pa}}{{I}_{pc}}=1$ at every scan rate. In the presented cyclic voltammogram, the peak heights are (roughly) the same, thus suggesting a good reversibility of lithium insertion into and extraction from nLTO. In ideal systems it is also verified that the peak potential separation, Δ*E*_*p*_, is kept constant independently of the applied scan rate. Clearly, this is not the case for nLTO/SWCNT composites. As shown in Fig. 5a, at higher scan rates, significant over-potentials are required to drive the electrochemical processes, leading to electrode polarization (quasi-reversible process)^33, 35^. Finally, cyclic voltammetry can also be used to characterize the capacitive mechanisms in a system by considering the following equation^36, 37^.

1 $${i}_{p}=a{\upsilon }^{b}$$

where *i*_*p*_, *a*, $\upsilon$ and *b* are the peak current, a constant, the scan rate and an exponential factor, respectively. Therefore, *b* states how the peak potential progresses upon increasing scan rate. Usually, this parameter equals 0.5 for diffusion-limited processes and 1 for surface-redox processes^36–38^. Equation (1) can be re-arranged as:

2 $$log({i}_{p})=b\mathrm{.}log(\nu )+b\mathrm{.}log(a)$$

and therefore, *b* can be easily determined from the slope of log (*i*_*p*_) against log(*ν*), as shown in Fig. 5b. As the slope is 0.55, it is possible to assume that the lithium insertion/extraction in nLTO is a mass-diffusion limited process^9, 35, 38^.

The high rate capability of nLTO/SWCNT composite electrodes, was studied by galvanostatic charge-discharge experiments (Fig. 5c) at different current rates from 0.1C (3.1 *μ*A.cm^−2^) to 100C (3.3 mA.cm^−2^). The charge/discharge curves present a flat plateau around 1.55 V, corresponding to the two-phase equilibrium between Li_4_Ti_4_O_12_ and Li_7_Ti _5_O_12_. This feature, plus the narrow gap between the charge and discharge plateaus usually indicates a good Li^+^ insertion/extraction kinetics^39, 40^. Upon increasing the current rate, these features are still kept and only above 30C does the gap between plateaus become significant, indicating a pronounced polarization effect^33^. These observations are in good agreement with the previous cyclic voltammetry analysis. It is also possible to observe that after the plateau stage an additional capacitive and irreversible process takes place. This is most probably associated to the insertion of lithium ions in the carbon network. The same behavior has been observed for similar systems, as reported by Sun *et al*.^41^. However, it has been previously demonstrated that the contribution of the carbon nanotubes to the total capacity can be neglected after the first cycles^36^. It is also possible that the charge-discharge curves in Fig. 5c exhibit a pronounced sloping outside the plateau region, suggesting a considerable capacitive contribution to the total charge storage, due to faradaic reactions with surface atoms^10^. It was also assumed that these sloping voltages would result from the strain between the poor-Li and rich-Li phase interfaces^42^. At small scales, where the amount of atoms near the interface is larger, different unit cell volumes lead to an energy penalty and reduced voltage plateaus. However, LTO is known as a zero strain material^42^. Therefore, this approach should not be suitable for the system under study^9, 42^. For smaller LTO particles it was observed that a distribution of different redox potentials at the near surface *vs* bulk leads to extra capacity storage and sloped voltages. This effect was verified for particles quite smaller than LTO. However, systems with increased surface areas can as well exhibit sloping voltages and reduced flat plateau regions^9^. The capacity measured by galvanostatic charge-discharge experiments as a function of the cycle number is shown in Fig. 5d. A maximum capacity as high as 173 mAh.g^−1^ was obtained for the composites at 0.1C (3.1 *μ*A.cm^−2^). It is clear that the capacity decreases upon increasing current, revealing the impact of the kinetic limitations in the overall nLTO/SWCNT electrochemical performance. However, even at 100C (3.3 mA.cm^−2^), the nLTO/SWCNT electrodes still retain a relatively high capacity of 70 mAh.g^−1^. In comparison, nLTO capacity (without any type of conductive additive) has decreased to virtually zero at 10C (1.9 mA.cm^−2^). At this rate, the nLTO/SWCNT composite still presents 50% of its initial capacity (roughly). The good specific capacity and high reversibility of the composites might be attributed to the small size of the nLTO particles and to the high electrical conductivity of SWCNT, as this is not observed for bulk LTO (see Supplementary Fig. S7). In first place, smaller particles lead to an optimized utilization of the active material and most importantly to shorter diffusion paths for both electrons and lithium ions^7^. The small size of the particles might lead as well to some additional pseudocapacitive effects, resulting in an optimized capacity. As the SWCNT compensate for the poor electrical conductivity of nLTO, the composites still present a good performance even at high rates. Moreover, the carbon nanotubes may also increase the porosity of a composite and therefore the transport of electrolyte and lithium ions through the network is highly facilitated^30^.

Finally, the cycling performance of nLTO/SWCNT composites was studied after 1000 cycles at 1C (3.30 *μ*A.cm^−2^) and the results are shown in Fig. 5e. A Coulombic efficiency of 98.5% was obtained for the first cycle, due to irreversible capacity. This parasitic capacity decreases with increasing cycle number and at around 100 cycles, the Coulombic efficiency stabilizes around 99.8%. A relatively high capacity of ~136 mAh.g^−1^ is retained up to 1000 cycles at 1C. Most probably, the high crystallinity of nLTO and stability of nLTO/SWCNT composites leads to the good cycling behavior represented in Fig. 5e. It is important to stress the fact that no other electrodes with different mass loading and thickness were tested in this work. This paper only focus in the validation of the proposed methodology. Further testing and optimized devices will be considered for further publications.

## Discussion

In Table 1 it is summarized the most important electrochemical features of nLTO/SWCNT composites. A direct comparison with literature references is not possible due to the several different ways that electrodes can be prepared and tested. However, the composites prepared by ultrasonic spray deposition can be placed in a broader electrochemical context and some comments can be drawn. Haetge *et al*.^7^ prepared LTO thin films (50 ± 2 *μ*g.cm^−2^) by a soft-templating route, achieving a capacity of 155 mAh.g^−1^ at around 30C (92 *μ*A.cm^−2^). However, even at 64C (184 *μ*A.cm^−2^) the thin films capacity is kept around 148 mAh.g^−1^ while exhibiting a highly stable cycling performance. Some of this optimal properties might arise from the fact that the thin films used are made out of very small particles. Once again, it seems that one key point for manufacturing LTO electrodes with very high rate capability is the preparation of highly porous nanocrystalline structures^7^. However, particle size must be taken into consideration as well. According to Kavan *et al*.^43^ for particles with surface areas less than 20 m^2^.g^−1^, the charge capability is roughly proportional to the logarithm of the particle surface area. Materials with surface areas up to 100 m^2^.g^−1^ present the theoretical capacity of LTO, when charged/discharged at different current rates. However, for very small particles (surface area >100 m^2^.g^−1^) the rate capability decreases considerably, due to a decrease in the lithium ion diffusion coefficient^42, 43^. Other spinel Li_4_Ti_5_O_12_ thin films, prepared by electrostatic spray deposition, present a relatively good coulombic efficiency and a capacity of 155 mAh.g^−1^at C/18^44^. Table 1 suggests a much better electrochemical performance for the composites under study. It is important to stress the fact that both thickness and mass load of these devices are not mentioned and no conductive additives were added to the film. Nevertheless, at such low scan rate, even bare nLTO presents a higher specific capacity (~175 mAh.g^−1^). Moreover, these thin films may not present important or direct applications due to their very low mass load of active material. In the micrometer range, LTO electrodes prepared by ink jet printing, with an average thickness of (1.7~1.8 *μ*m), present a capacity of 153 mAh.g^−1^ (300^*th*^ cycle), at a current of 10.4 *μ*A.cm^−2^ ^45^. In spite of being quite similar to the results obtained for nLTO/SWCNT composites under the same conditions, the coulombic efficiency of the ink jet printed devices is lower (around 98%). Still in the same thickness range, LTO/CNT composites prepared by extrusion^46^ and template-based solution route^47^ present capacities of 142 mAh.g^−1^ (1.75 A.g^−1^) and 81 mAh.g^−1^ (17.5 A.g^−1^), respectively. All the examples aforementioned show that composites of nLTO and carbon nanotubes, manufactured by a combination of liquid phase processing and ultrasonic spray deposition, can be competitive anodes for large scale industrial applications. As the carbon nanotube networks improve the mechanical properties of the overall electrode, the nLTO/SWCNT composites might become of major importance for flexible technology, namely in microbatteries or supercapacitors applications. In spite of it being possible to prepare nLTO dispersions in a very simple and easy way, the mechanism behind this process is not fully clear. In fact, the ultrasound treatment here described has been developed as an effective tool for the delamination of layered crystals, where their structure is kept by weak van der Waals forces^19, 21, 23^. However, Li_4_Ti_5_O_12_ does not present the typical layered material structure, thus, nLTO formation mechanisms must differ from the ones previously described^18, 19, 21^.

**Table 1:** Summary of the electrochemical properties of nLTO/SWCNT composites. The electrodes presented a LTO mass load of 200 *μ*g.cm^2^ and a thickness of 1 *μ*m.

C-Rate	Current Density (*μ*A.cm^−2^)	Specific Capacity (mAh.g^−1^)
0.1	3.3	173
0.5	16.5	140
1	35	130
5	175	126
10	350	110
30	1.0 × 10^3^	100
50	1.8 × 10^3^	82
100	3.5 × 10^3^	70

It is possible that the ultrasound irradiation leads to the fragmentation of particle clusters already present in the raw powder. In fact, small powder particles can easily agglomerate via weak electrostatic interactions. It is also possible that particles are held together by strong attractive forces, thus forming aggregates or “hard agglomerates”^48^.

**Figure 6:**
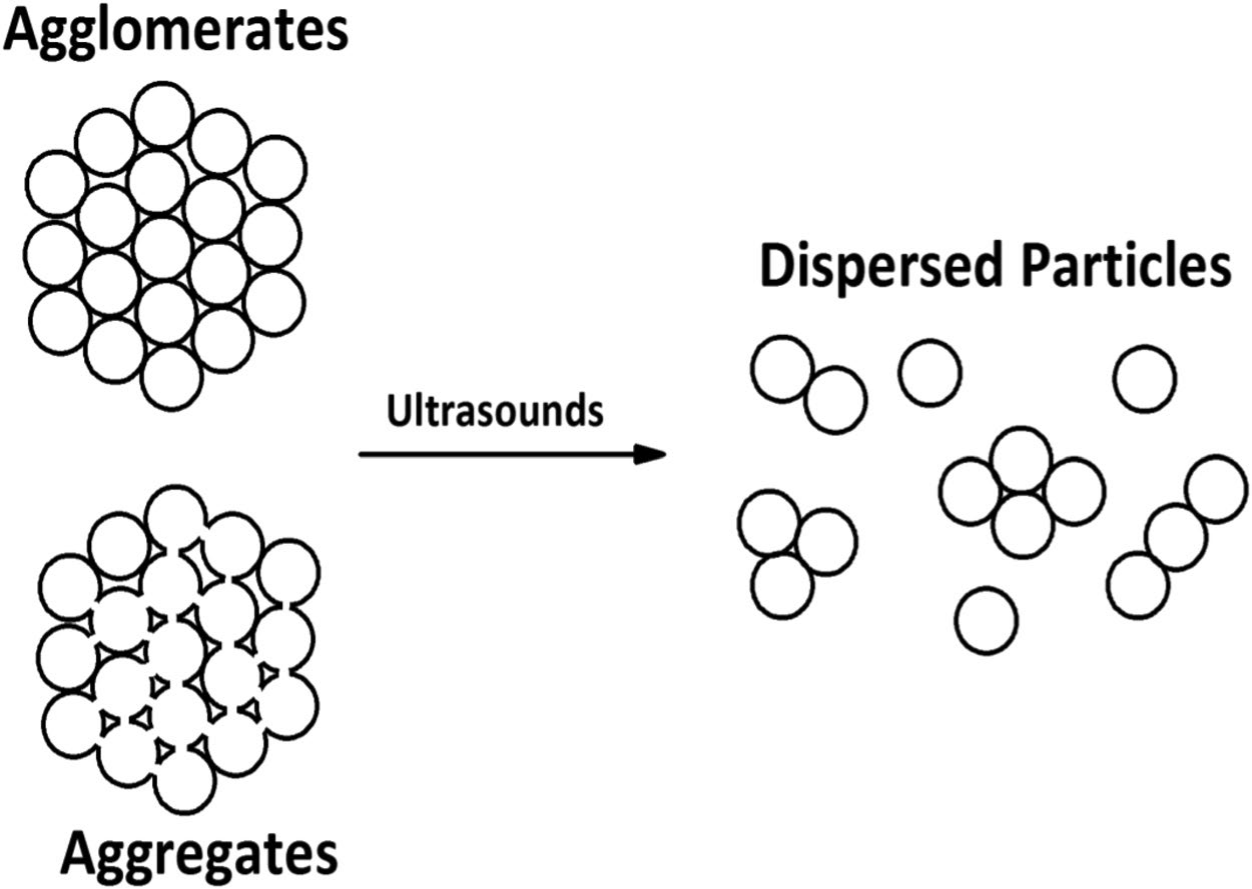
Possible formation mechanism for nLTO particles^48^.

Upon ultrasound irradiation these clusters are easily fragmented into smaller structures or even single particles, as shown in Fig. 6. In the case of agglomerates, the high energy events resulting from a cavitation bubble burst is powerful enough to overcome the weak interactions between the particles. Therefore, it is possible to separate particles individually. This is usually referred to as an erosion process. Agglomerates on the other hand, require structural imperfections and/or surface defects where fracture can initiate. While defects can be found in the aggregate, it will be continuously broken down into smaller structures^48^. In principle, both processes are behind the ultrasound assisted preparation of titanium dioxide^48^ silica^49^ and alpha-alumina dispersions^50^ among others. In fact, before assisting the exfoliation of layered crystals^18, 51^ ultrasounds have been widely used to debundle and disperse carbon nanotubes^52^. Based on this assumption, it is likely that upon sonication large clusters of LTO are broken down into smaller structures.

**Figure 7:**
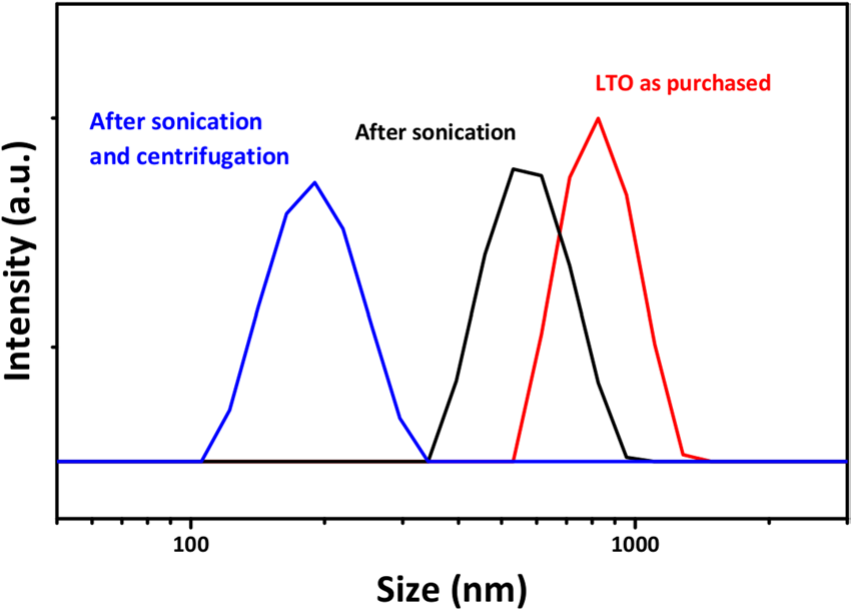
Particle size distribution for LTO (**a**); LTO after sonication without centrifugation (**b**) and nLTO (**c**).

This hypothesis might be supported by DLS measurements (Fig. 7). Initially, the average size of LTO particles is around 800 nm. Upon sonication, the clusters or particles are fragmented and the average LTO size is reduced to 680 nm. The particle size distribution had also shrunk by a factor of 3 (approximately). Finally, after centrifugation a nLTO dispersions composed of particles with an average size of 174 nm is obtained.

**Figure 8:**
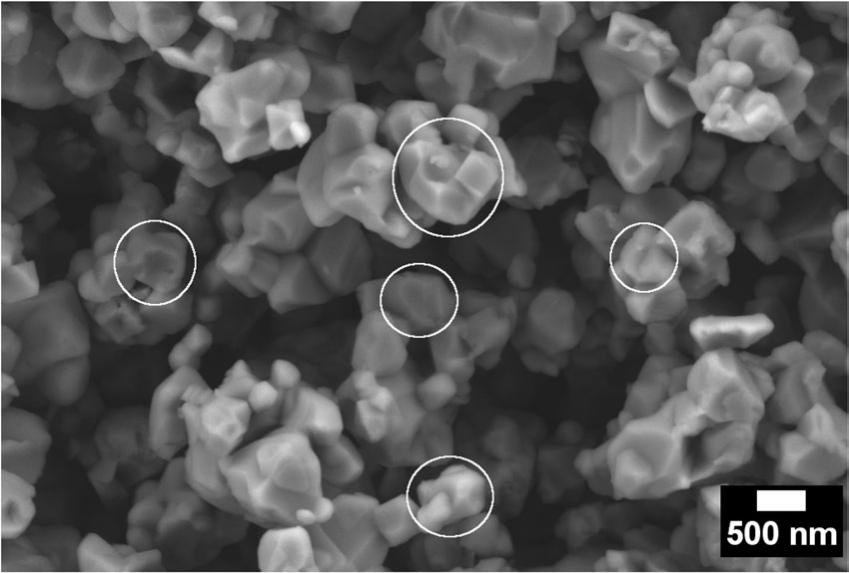
Non-dispersed LTO particles revealing possible surface cleavage or fracture sites.

It might also possible that nLTO dispersions are composed at some extension by particles resulting from the high energy phenomena and high velocity inter-particles collisions processes that occur in a system under high power ultrasound irradiation^53–55^. In order to verify this hypothesis, the non-dispersed LTO precipitate was collected after centrifugation and analyzed under SEM (Fig. 8). The average particle size does not seem to be heavily affected by the ultrasound irradiation. However, the morphology of the particle has suffered some clear modifications. The clear sharp edges and flat surfaces previously observed (see Supplementary Fig. S1) can no longer be seen. Moreover, surface locations that seem to be breaking or fracture points are new morphological feature of the LTO particles. Some of these were marked with white circles in Fig. 8. These findings support the idea that inter particle collision and high energy microjets might as well contribute to the formation of nLTO. However, some additional experimental validation is need to fully support this hypothesis.

To summarize, in this work it was presented for the first time the preparation of nano Li_4_Ti_5_O_12_ particles (average size (174 nm) by a liquid-phase sonication procedure. After the initial solvent study, highly stable suspensions of nLTO in IPA were obtained with a concentration of 0.1 mg.mL^−1^. Percolation studies on nLTO/SWCNT electrodes prepared by ultrasonic spray deposition revealed that a very small amount of conductive additive (~0.15% wt) is required in order to establish the very first conductive pathways. However, a mass fraction of ~15% is needed to maximize the electrochemical utilization of nLTO/SWCNT composites. For this mass fraction of carbon nanotubes a maximum capacity of 173 mAh.g^−1^ was obtained at 0.1C (3.1 *μ*A.cm^−2^) for electrodes presenting a nLTO mass load of 198 *μ*g.cm^2^ and 1110 nm thick. The composites still retain a specific capacity as high as 70 mAh.g^−1^ at 100C (3.3 mA.cm^−2^). Further electrochemical characterization showed that composites are also highly stable and present good chemical reversibility over cycling. After 1000 cycles at 1C (33.0 *μ*A.cm^−2^) the specific capacity stabilizes at around 136 mAh.g^−1^ and the Coulombic efficiency at 99.5%. The sonication process here presented allowed the preparation of lithium titanate nanoparticles with enhanced electrochemical properties. Moreover, it opens the possibility to future research work focused on the development of nanoparticles from other bulk non-layered materials. It is also shown that carbon nanotubes are indeed of major importance for the optimization of an electrode’s electrochemical performance. Finally, it was shown again that liquid phase processing followed by ultrasonic spray deposition is a cheap and simple route for the manufacturing of nanomaterials based electrodes, with the possibility of being implemented in large scale applications. Therefore, such combination might become a powerful way of preparing optimal electrodes or devices for several industrial or technological applications.

At the moment, the use of nLTO/SWCNT composites as the negative electrode of hybrid supercapacitors (against activated carbon) is being considered. They might be also used as anodes for high-power batteries. Some preliminary experiments have shown that it is possible as well to process olivine lithium iron phosphate by ultrasound irradiation. Therefore, a full battery nLTO/nLFP (nano lithium iron phosphate) might be of interest for future investigation.

## Experimental Section

### nLTO and SWCNT dispersion preparation

Li_4_Ti_5_O_12_ powders were processed in a FisherBrand 11207 sonic bath. Initially, 2 mg.mL^−1^ suspensions were prepared in acetone, benzyl alcohol, benzyl benzoate, cyclohexanone, dibromomethane, N,N Dimethylformamide (DMF), isopropyl alcohol (IPA), N-methyl-2-pyrrolidone (NMP) and water. After being under ultrasonic treatment for 3 hours at 37 kHz and 200 W power, the suspensions were centrifuged at 1500 rpm for one hour in a Heraeus Multifuge x1 Centrifuge. In order to estimate the quantity of the material dispersed the absorbance per cell length, *A/l*, of these dispersions was measured at 500 nm in a Varian, Cary 6000i UV-vis-NIR spectrometer. All measurements were performed in 10 mm matched quartz cuvettes. The solvents were then tested at different initial concentrations (5 mg.mL^−1^ and 10 mg.mL^−1^), different frequencies (37 kHz and 80 kHz). The same optical measurements were repeated for these samples. The concentrations were confirmed by filtering 100 mL of dispersion in alumina membranes (pore size ~20 nm) and weighing the retained mass. Optimized dispersions in water (10 mg.mL^−1^; 3 hours, 37 kHz and 200 W) were then centrifuged at 5000 rpm for 30 mins in order to precipitate the nLTO and replace the water with IPA. 0.1 mg.mL^−1^ dispersions of SWCNT were prepared by dispersing 10 mg of P3-SWCNT in 100 mL of IPA for one hour in a Fisherbrand Ultrasonic Dismembrator (30 W, 40% amplitude). All used solvents, raw LTO and SWCNT were sourced from Sigma Aldrich (99.99% purity), Linyi Gelon LIB Co., Ltd. and Carbon Solutions, Inc, respectively. All chemicals were used as purchased.

### Structural and morphological characterization

X-ray diffraction (XRD) was performed in a fully automated Bruker D5000 powder diffractometer equipped with a monochromatic Cu K*α* radiation source (*λ* = 0.15406 nm) and a secondary monochromator. XRD patterns were collected between 10° < *θ* < 80°, with a step size of 2*θ* = 0.05° and a count time of 12 s/step. All the samples were supported on a single crystal of silicon. Scanning Electron Microscopy (SEM) was performed in a Zeiss Ultra Plus microscope in high vacuum mode and with an acceleration voltage of 5 keV. Raman spectra were recorded at room temperature using a Witec Alpha 300 system with a laser excitation wavelength of 532 nm. A laser power of ~0.4 mW was used with a 20x objective lens. Representative spectra were acquired for each sample by averaging 10 distinct spectra, each with an acquisition time of 30 s. X-ray photoelectron spectroscopy (XPS) was performed in an ion pumped VG Microtech CLAM 4 MCD equipment using a 200 W unmonochromated Mg X-ray excitation source (1253.6 eV), with samples supported on silicon substrates. The analyser was operated at constant pass energy of 100 eV for wide scans and 20 eV for detailed scans. The XPS spectra were analyzed and fitted using CasaXPS software and spectra calibration was done fixing the position of the C1s = 284.9 eV as reference. BET isotherms were acquired in a Quantachrome Nova 4200e station using nitrogen at 77 K. The specific surface area was obtained by applying the BET equation to the linear portion of the isotherm curves. The pore size and volume were determined by using the BJH method in both adsorption and desorption isotherm curves. All samples were outgassed in nitrogen atmosphere at 100°C for 16 hours before measurements. The remaining morphological characterization was done in a FEI Titan Transmission Electron Microscope (TEM) operated at 300 keV in bright field configuration.

### Electrode manufacture and characterization

Slurries were prepared by mixing 80% of active material, 10% PVDF and 10% active carbon. In a normal procedure, proper amounts of active material and carbon addictive were very well mixed in a mortar and added to a 4 mL solution of PVDF in NMP. After mixing for 24 hours, the resulting slurries were pasted in 2.54 cm^2^ copper substrates and allowed to dry overnight at 85 °C. The processed materials were sprayed onto copper substrates (2.54 cm^2^) in a USI Prism Ultracoat 300. Briefly, suspensions of nLTO/SWCNT were fed into an automated pumping system that delivered the suspension at the tip of an ultrasonic spray head at a flow rate of 1 mL.min^−1^. Ultrasonic vibrations disintegrated the liquid into small drops producing a mist that was propelled away from the spray forming tip by a low pressure air (69 kPa) stream in the form of a spray cone. The mist was deposited onto substrates attached to a hot plate that maintained a temperature suitable for evaporation of the solvent The spray head was moved at a constant spray height (35 mm) and speed (100 mm.s^−1^) by an automated gantry in *x* and *y* directions drawing a previously designed and specific spray pattern that produced a uniform film. Electrode thickness was measured by profilometry using a Dektak 6 M, Veeco Instruments. Electrical conductivity values were determined using a four-point probe technique. The resistivity was measured with a Keithley 2400 source meter (Keithley Instruments, Inc.) and LabView interface (National Instruments, Inc.).

### Electrochemical characterization

EL-CELL were used to assemble the electrodes in an argon filled gloved box by using lithium foil as counter electrode, lithium wire as reference and 1 M LiFP_6_ in a EC:DMC (50:50) as electrolyte. Cyclic voltammetry and galvanostatic charge-discharge experiments were performed in a potential range of 1.2 to 2 V and 1.2 to 3 V vs Li^+^/Li, respectively. All the measurements were performed in a BioLogic VMP 300.

## Electronic supplementary material

Supplementary Material
