# Supplementary material for: Lithium Titanate/Carbon Nanotubes Composites Processed by Ultrasound Irradiation as Anodes for Lithium Ion Batteries
Source: Sci Rep. 2017 Aug 8;7:7614. doi: 10.1038/s41598-017-06908-3 (PMC5548767; doi:10.1038/s41598-017-06908-3)
Supplement: Supplementary file 1 — Supplementary Material [file 41598_2017_6908_MOESM1_ESM.pdf]

Lithium Titanate/Carbon Nanotubes Composites Processed by Ultrasound Irradiation as Anodes for Lithium Ion Batteries

Electronic Supplementary Information

João Coelho,[1] Anuj Pokle,[2], Sang-Hoon Park[1], Niall McEvoy[1], Nina C. Berner,[1], Georg S. Duesberg[1,3] and Valeria Nicolosi*[1,2]

[1] *School of Chemistry/CRANN, Trinity College Dublin, College Green, Dublin 2, Ireland.*
[2] *School of Physics/CRANN, Trinity College Dublin, College Green, Dublin 2, Ireland.*
[3] *Institute of Physics, EIT 2, Faculty of Electrical Engineering and Information Technology, Werner-Heisenberg-Weg 39 , 85577 Neubiberg, Germany*
E-mail: coelhoj@tcd.ie; nicolov@tcd.ie

SEM of Raw Materials:

The LTO used throughout this work was purchased from Linyi Gelon LIB Co., Ltd. As there was not an evident advantage on synthesising lithium titanate in the laboratory, a commercial source was used instead.

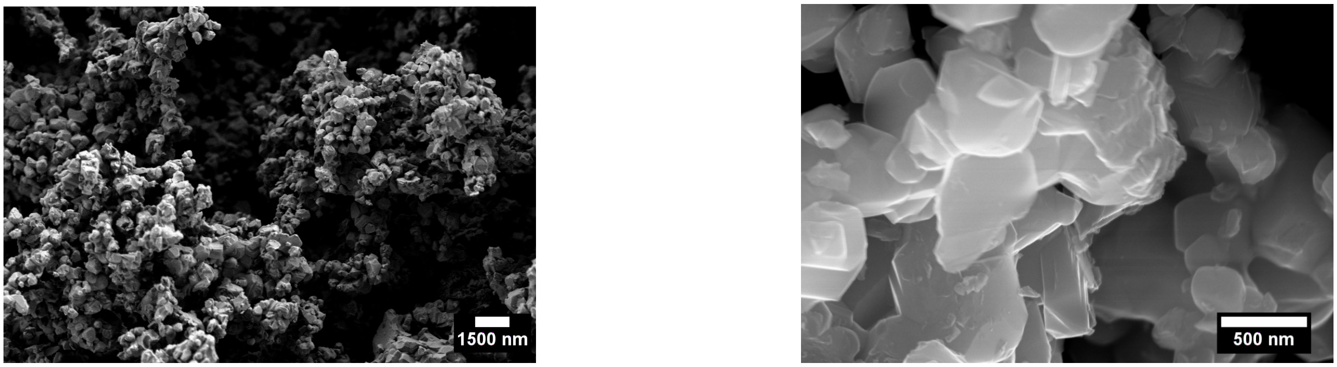

(a) (b)

Figure S1. SEM a) secondary electrons and b) In-lens micrographs of LTO before ultrasound treatment.

Fig. S1a and Fig. S1b show two micrographs of the purchased LTO as received without any type of treatment. As described in the main manuscript the average particle size before ultrasonication is 800 nm. In terms of morphology, this sample is composed by particles exhibiting very well defined edges and flat surfaces. Regarding dimensions, these particles size present a large size distribution in the micrometer range. From the SEM micrographs it was not possible to assess any other relevant morphological features.

Raman and XPS Analysis:

Fig. S2 depicts the Raman spectra of unprocessed and liquid phase processed LTO. Both spectra show peaks at 235, 274, 350, 428 and 675 cm^{-1}, which are characteristic of spinel LTO.[1] In a very simple way, the peak at 235 cm^{-1} is usually associated with the bending vibrations of O-Ti-O bonds,[2] at 428 and 350 cm^{-1} the stretching vibrations of Li-O in the LiO_4 and LiO_6 polyhedra occur, respectively,[2,3] whilst the

higher frequency bands correspond to the vibrations of Ti-O bonds in the TiO_6 octahedra. According to literature, these are the five bands characteristic of spinel $Li_4Ti_5O_{12}$ corresponding to the Raman-allowed phonon peaks (A_{1g}+E_g + $3F_{2u}$).[2,4] The presented Raman data is in good agreement with reference works such as Aldon *et al*[5] and Julien *et al.*[4] However, some authors, also reported the existence of an additional peak around 160 cm^{-1} that should correspond to the bending vibration of O-Li-O bonds.[2,6] The broad shoulder around 750 cm^{-1} has been attributed to the vibrations of Ti-O bonds in the TiO_6 octahedra as well (F_{2u} mode).[1,7,8] It is clear that LTO Raman spectrum is not fully understood yet. Nevertheless, it has been commonly accepted that $Li_4Ti_5O_{12}$ phases can be identified by assigning the main five characteristic bands.[2,7,8] The nLTO spectrum also exhibits a very strong peak around 550 cm^{-1}. This signal arises from the silica substrate upon which nLTO was deposited. LTO was not deposited into a substrate, but compressed into a pellet instead. Therefore, it does not exhibit any signal at 550 cm^{-1}.

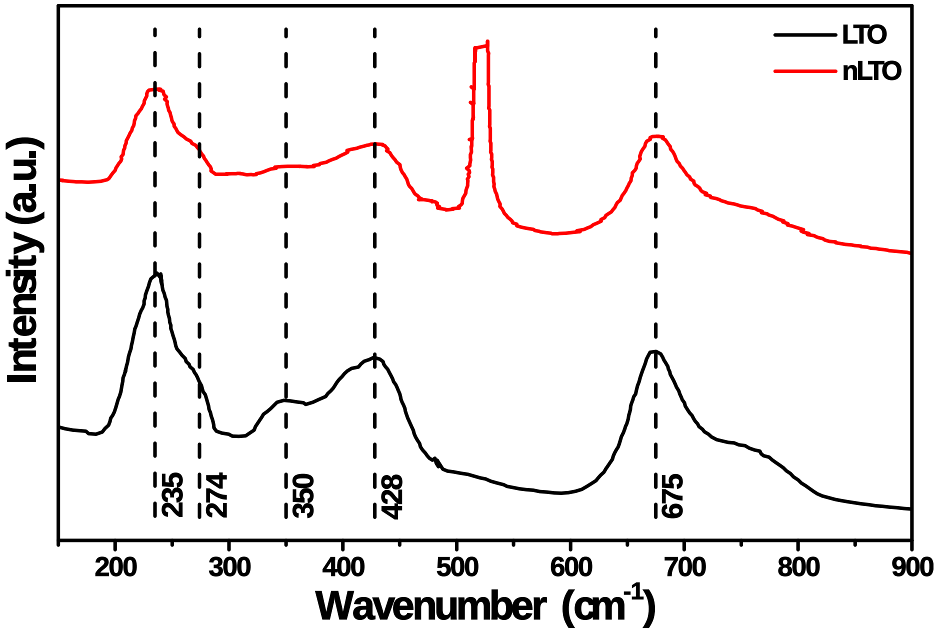

Figure S2. Raman spectra of LTO and nLTO samples. Vertical dashed lines are used to compare peak position across samples. The 550 cm^{-1} on nLTO spectrum has its origin on the silica substrate.

In order to further characterize the nLTO particles' surface, XPS measurements were carried out. The XPS survey scan presented in Fig. S3.a allowed the identification of four main elements. The C1s signal has its origin in amorphous carbon (adventitious carbon) with a variety of C-C bonds, formed in all surfaces exposed to air.[9] The Ti2p (Fig. S3.b) spectrum revealed a doublet composed of two peaks, Ti2$p_{\frac{3}{2}}$ (458.61 eV) and Ti2$p_{\frac{1}{2}}$ (464.31 eV). Usually, this signal is associated with a titanium IV oxidation state in an octahedral environment, in good agreement with the aforementioned Raman characterization.[10,11] A very small amount of Ti^{3+} could also be detected.[7,12] The peak at 529.95 eV in the O1s spectrum (Fig. S3.c) is assigned to the Ti-O bond in LTO. We can still detect a pronounced peak at 531.99 eV associated with water in the sample and probably some carbon contamination.[10,13,14] The small peak at 532.61 eV is most probably associated with the silicon substrate. Finally, the Li1s peak at 54.61 eV is also characteristic of spinel LTO (Fig. S3.d).[13–15] Both Raman and XPS results corroborate the fact that nLTO is in fact a pure phase lithium titanate sample.

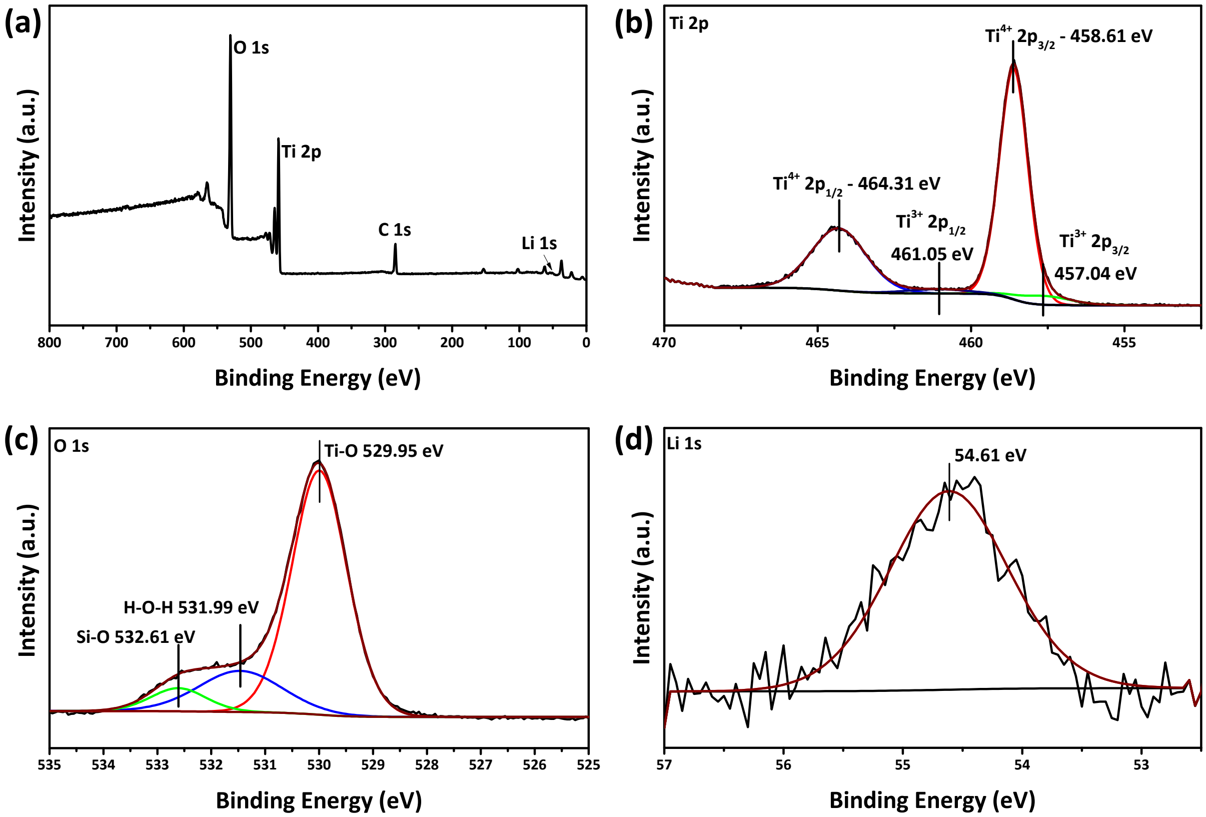

Figure S3. nLTO XPS survey spectrum (a), Ti2p (b), O1s (c) and Li1s (d) core-level spectra.

Percolation Studies on nLTO/SWCNT Composites

SWCNT are known to be percolative agents, *i.e*, there is a minimum fraction of carbon nanotubes (percolation threshold) that increases a system conductivity by several orders of magnitude. For example, the electrical conductivity of exfoliated MoO_3 loaded with 3% wt carbon nanotubes, increases eight orders of magnitude, from 10^{-6} to 10^{2} $S.m^{-1}$.[16] A similar trend is observed for exfoliated MoS_2 mixed with 2.6% wt carbon nanotubes.[17] In order to understand this type of behaviour, it is important to recall percolation theory. As all of the aforementioned materials (and nLTO as well) present a very low electrical conductivity, their composites with SWCNT can be regarded as a conductor-insulator mixture. In this case, according to percolation theory, the conductivity of the system can be described by:[18,19]

$$\sigma = \sigma_0(\phi - \phi_p)^n, \quad \phi > \phi_p \tag{1}$$

Equation 1 states that above the percolation threshold, ϕ_p, the electrical conductivity, σ, of a system depends firstly on the conductivity, σ_0, of the nanotubes. However, the sample will not be conductive if the amount, ϕ, of nanotubes is not above the percolation threshold, ϕ_p. In a very simple way, this threshold quantifies the minimum amount of SWCNT required to establish a conductive path from one edge of the electrode to the other opposite edge. Obviously, SWCNT are randomly distributed in the sample, until ϕ_p is achieved and the first conductive path is established. This idea implies that σ and ϕ are not proportional, as a higher mass fraction of carbon nanotubes does not necessarily imply a conductive system. In fact, immediately above the percolation threshold, only a very small amount of carbon nanotubes will contribute to the sample conductivity, while the rest can be considered "dead ends".[18] On the other hand, for the case where $\phi \gg \phi_p$, as it might be expected, all the sites in the sample will be in contact with SWCNT and therefore the sample will reach the conductivity (or at least in a good approximation) of SWCNT. In order to experimentally verify this idea and estimate the percolation threshold for nLTO, several electrodes with different SWCNT mass fractions were manufactured by ultrasonic spray deposition and their electrical conductivity measured by the four point probe method. The results are shown in Fig. S4. It is clear that the conductivity increases several orders of magnitude arising with a very low mass fraction of SWCNT, reaching 100 $S.m^{-1}$ for a nanotube load of 10%. As previously mentioned, this sharp increase in conductivity is usually associated with the formation of the first conductive pathway above ϕ_p and it is in good agreement with percolation theory.[16,20] Fitting experimental data with Equation 1 resulted in a $\phi_p \sim 0.15\%$ for nLTO. This value is much lower than the percolation thresholds estimated for other exfoliated materials/SWCNT composites ($\phi_p \sim 3\%$ for MoO_3,[16] and 2.6% for exfoliated MoS_2[17]). However, the geometry of the system, particle size, electrical properties and dimensions of SWCNT should be taken into consideration. For instance, polymeric nanoparticles/SWCNT systems present a ϕ_p of 0.12%.[21] A direct comparison across different systems might not possible due to the effect of several variables. Therefore, and in spite of some experimental error associated, the obtained ϕ_p for nLTO/SWCNT should be valid. Regarding the critical exponent, n, some remarks should be made as well. It is commonly accepted that it should be around 1.6 (two dimensional systems). However, more recently, exponents as high as 3 have been theoretically predicted and experimentally determined.[19] This confusion arises from the fact that most of the percolative systems can be (and in fact they are most of the times) modelled by a *discrete-lattice* approach. However, other models, such as the *continuum* system model, show that n might assume other values rather than the one universally accepted. More detailed information about percolative networks can be found elsewhere.[22]

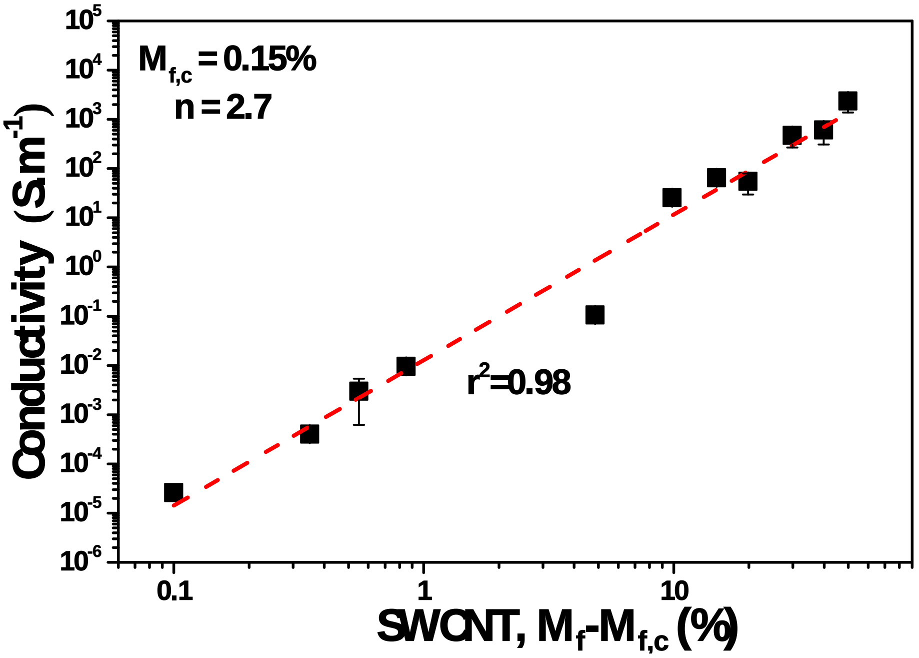

Figure S4. Electrical conductivity of thin films (∼2 μm thick) of nLTO/SWCNT composites plotted as a function of nanotube mass fraction. Usually this type of experiment should be conducted using the volume fraction of SWCNT and not the mass fraction. However it has been shown that for the used mass fractions the approximation is still valid.[16] The lines represent fits to percolation theory (Equation 1).

Composites Optimization

The total composites capacity results from the contribution of both nLTO and carbon nanotubes. Unless, additional effects are present, the specific capacity of nLTO is expected to be 175 mAh.g^{-1}. Regarding, SWCNT capacities as high as 1000 mAh.g^{-1} have been reported. However, at potentials above 1V versus Li^+/Li, SWCNT capacity is severely reduced.[23] Therefore, in the current work, the contribution of carbon nanotubes to the composites total capacity was estimated by charge discharge experiments, as shown in Fig. S5.

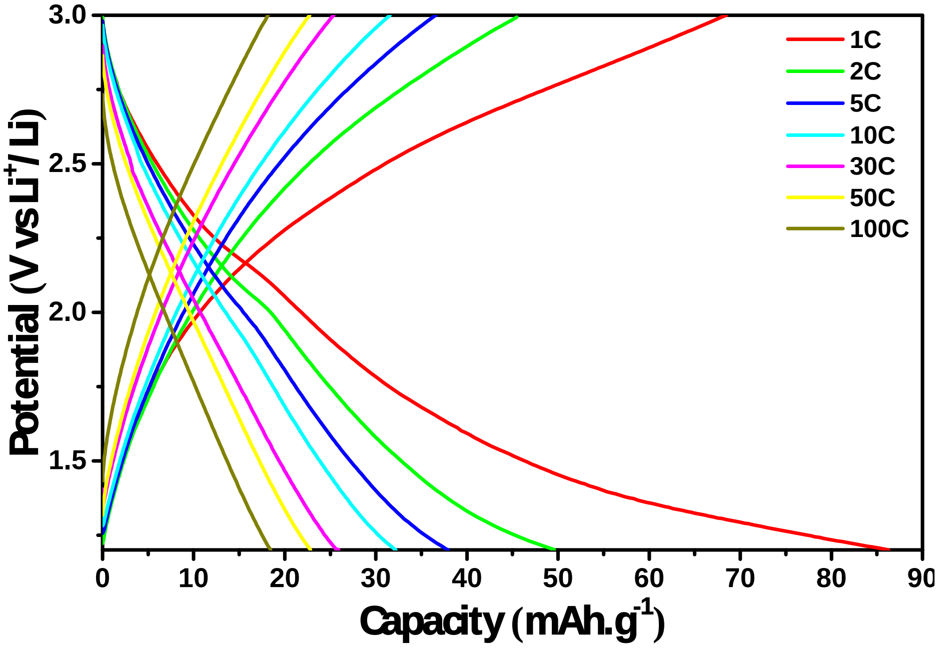

Figure S5. SWCNT capacity dependence on the applied current rate. The SWCNT electrodes were tested in the same conditions as the composites.

A maximum discharge capacity of 86 mAh.g^{-1} can be obtained at 1C. However, for the optimized electrodes with carbon nanotubes mass fractions of roughly 15%, SWCNT contribution for the total composite capacity should be relatively low. Therefore, it is possible to say that the nLTO is practically the only composite component that contributes to the total capacity.

Fig. S6 shows the composites specific capacity dependence on the carbon nanotube mass fraction at different current rates. As described in the main document, a carbon nanotube mass fraction of approximately 15% wt results in an optimized composites specific capacity. However, in this figure it is shown in a clearer way, that the capacity progression with increased carbon nanotubes content does not follow a clear and defined trend, most likely due to some errors associated with the conducted experiments.

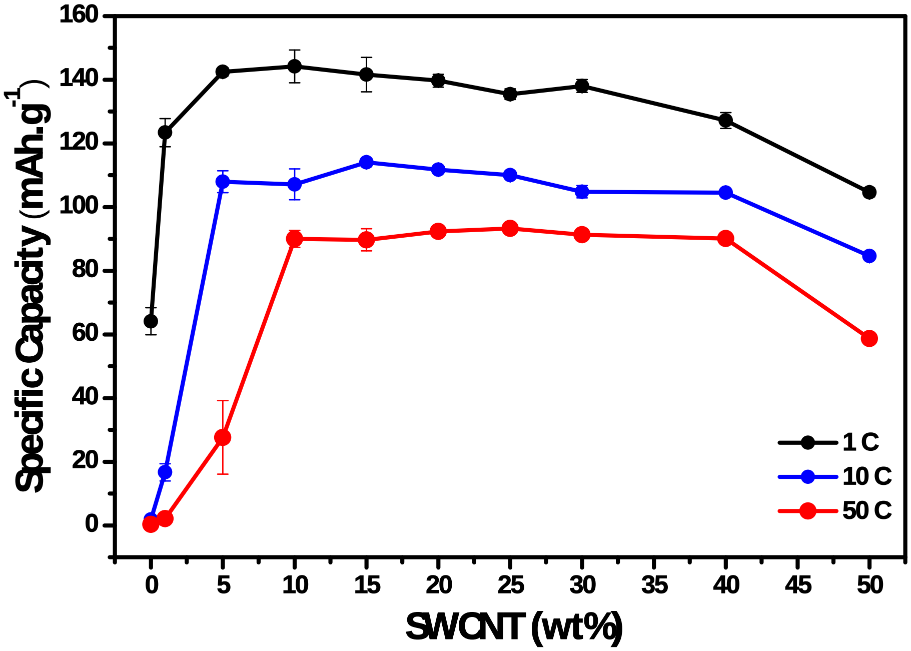

Figure S6. SWCNT capacity dependence on the applied current rate. The SWCNT electrodes were tested in the same conditions as the composites.

LTO Slurry Electrochemical Properties Overview

The characterization of LTO as purchased was performed by means of cyclic voltammetry (0.1 mV.s^{-1} - 10 mV.s^{-1}) and galvanostatic charge-discharge experiments (0.1C - 10C). In order to do so, LTO slurries (80%(wt) lithium titanate, 10%(wt) carbon black and 10%(wt) PVDF) were pasted into copper substrates.Then electrodes (0.9 mm diameter) were assembled in a half-cell configuration and tested against lithium foil in a 1M LiPF6 (EC:DEC (1:1)) electrolyte. The CVs obtained for LTO are represented in Fig. S7a.

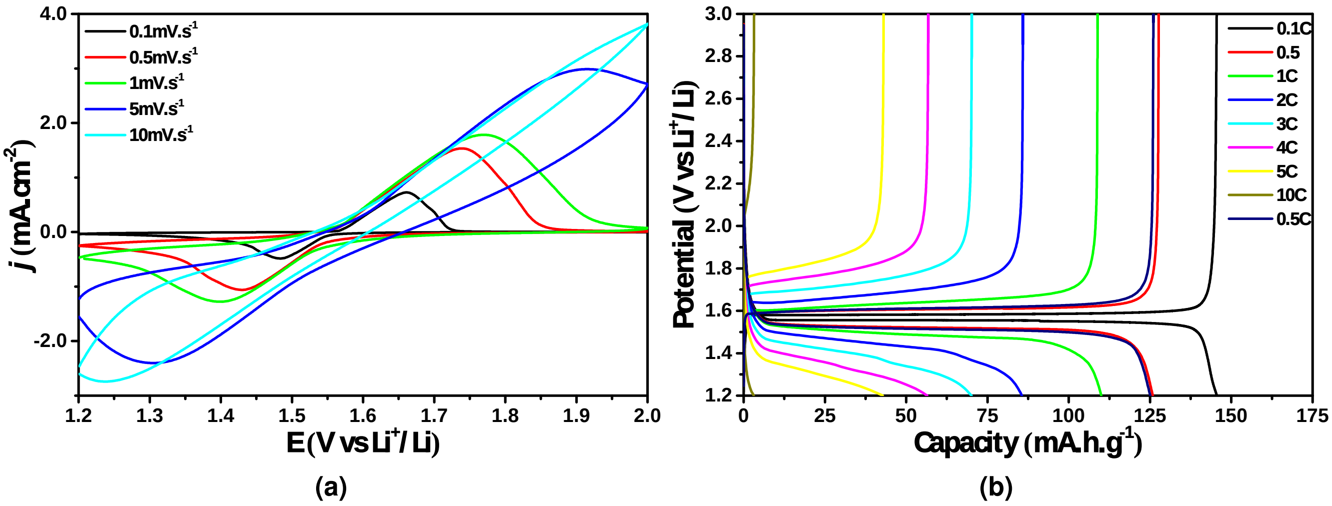

Figure S7. Lithium titanate a) cyclic voltammograms and b) galvanostatic charge-discharge curves at different currents.

The two characteristic peaks corresponding to the Ti^{3+}/Ti^{4+} redox couple are easily identified at low scan rate. However, when compared to the nLTO/SWCNT composites, LTO peaks are much broader, thus indicating a sluggish lithium insertion/extraction process. The slurry poor kinetics are more evident at high scan rates, where no clear peaks can be properly identified. A sloping of the anodic peaks upon increasing scan rate is also quite clear. Regarding the charge-discharge curves (Fig. S7b), it is possible to observe well-defined plateaus around 1.55 V, which are characteristic of lithium insertion/extraction in LTO. Moreover, this process seems to be quite reversible. However, the rate capability of bulk LTO is quite poor when compared to that of nLTO. The latter is capable of retaining 40% of its initial capacity at 100C, while LTO capacity falls to virtually zero at currents of only 10C.

The brief electrochemical characterization of LTO as purchased shows that the starting material used in this study is indeed conventional lithium titanate. Therefore, it is possible to assume that nLTO properties should only result from the processing method and not from the nature of the starting material.

References

1. Lee, D. K. *et al.* Synthesis of Heterogeneous $Li_4Ti_5O_{12}$ Nanostructured Anodes with Long-Term Cycle Stability. *Nanoscale Res. Lett.* **5**, 1585–1589 (2010).
2. Baddour-Hadjean, R. & Pereira-Ramos, J.-P. Raman Microspectrometry Applied to the Study of Electrode Materials for Lithium Batteries. *Chem. Rev.* **110**, 1278–1319 (2009).
3. Zhang, D. R. *et al.* Synthesis and Characterization of Nanocrystalline $LiTiO_2$ using a One-Step Hydrothermal Method. *J. Ind. Eng. Chem.* **13**, 92–96 (2007).
4. Julien, C., Massot, M. & Zaghib, K. Structural Studies of $Li_{4/3}Me_{5/3}O_4$ (Me= Ti, Mn) Electrode Materials: Local Structure and Electrochemical Aspects. *J. Power Sources* **136**, 72–79 (2004).
5. Aldon, L. *et al.* Chemical and Electrochemical Li-Insertion into the $Li_4Ti_5O_{12}$ Spinel. *Chem. Mater.* **16**, 5721–5725 (2004).
6. Yi, T.-F. *et al.* Structure and Physical Properties of $Li_4Ti_5O_{12}$ Synthesized at Deoxidization Atmosphere. *Ionics* **17**, 799–803 (2011).
7. Leonidov, I. *et al.* Structure, Ionic Conduction, and Phase Transformations in Lithium Titanate $Li_4Ti_5O_{12}$. *Phys. Solid State* **45**, 2183–2188 (2003).
8. Julien, C. & Zaghib, K. Electrochemistry and Local Structure of Nano-sized $Li_{4/3}Me_{5/3}$ O_4 (Me = Mn, Ti) Spinels. *Electrochim. Acta* **50**, 411–416 (2004).
9. Swift, P. Adventitious Carbon - The Panacea for Energy Referencing? *Surf. Interface Anal.* **4**, 47–51 (1982).
10. Shi, Y., Wen, L., Li, F. & Cheng, H.-M. Nanosized $Li_4Ti_5O_{12}$/Graphene Hybrid Materials with Low Polarization for High Rate Lithium Ion Batteries. *J. Power Sources* **196**, 8610–8617 (2011).
11. Zhao, Y., Liu, G., Liu, L. & Jiang, Z. High-Performance Thin-Film $Li_4Ti_5O_{12}$ Electrodes Fabricated by Using Ink-Jet Printing Technique and Their Electrochemical Properties. *J. Solid State Electrochem.* **13**, 705–711 (2009).
12. Wan, Z., Cai, R., Jiang, S. & Shao, Z. Nitrogen and TiN-Modified $Li_4Ti_5O_{12}$: One-Step Synthesis and Electrochemical Performance Optimization. *J. Mater. Chem.* **22**, 17773–17781 (2012).
13. Li, Y., Bettge, M., Bareño, J., Trask, S. E. & Abraham, D. P. Exploring Electrochemistry and Interface Characteristics of Lithium-ion Cells with $Li_{1.2}Ni_{0.15}Mn_{0.55}Co_{0.1}O_2$ Positive and $Li_4Ti_5O_{12}$ Negative Electrodes. *J. Electrochem. Soc.* **162**, A7049–A7059 (2015).
14. Pei, X. *et al.* Preparation and Characterization of Nanotube Li-Ti-O by Molten Salt Method. *Chem. China* **2**, 265–269 (2007).
15. Yang, Z. *et al.* Highly Reversible Lithium Storage in Uniform $Li_4Ti_5o_{12}$/Carbon Hybrid Nanowebs as Anode Material for Lithium-ion Batteries. *Energy* **55**, 925–932 (2013).
16. Hanlon, D. *et al.* Production of Molybdenum Trioxide Nanosheets by Liquid Exfoliation and Their Application in High-Performance Supercapacitors. *Chem. Mater.* **26**, 1751–1763 (2014).
17. Cunningham, G. *et al.* Percolation Scaling in Composites of Exfoliated MoS_2 Filled with Nanotubes and Graphene. *Nanoscale* **4**, 6260–6264 (2012).
18. Stauffer, D. & Aharony, A. *Introduction to Percolation Theory* (CRC press, 1994).
19. Pecharromán, C. & Moya, J. S. Experimental Evidence of a Giant Capacitance in Insulator–Conductor Composites at the Percolation Threshold. *Adv. Mater.* **12**, 294–297 (2000).

20. Higgins, T. M. *et al.* Effect of Percolation on the Capacitance of Supercapacitor Electrodes Prepared from Composites of Manganese Dioxide Nanoplatelets and Carbon Nanotubes. *ACS Nano* **8**, 9567–9579 (2014).

21. Jurewicz, I. *et al.* Locking Carbon Nanotubes in Confined Lattice Geometries - A Route to Low Percolation in Conducting Composites. *J. Phys. Chem. B* **115**, 6395–6400 (2011).

22. Feng, S., Halperin, B. & Sen, P. Transport Properties of Continuum Systems Near the Percolation Threshold. *Phys. Rev. B* **35**, 197 (1987).

23. Landi, B. J., Ganter, M. J., Cress, C. D., DiLeo, R. A. & Raffaelle, R. P. Carbon Nanotubes for Lithium Ion Batteries. *Energy Environ Sci.* **2**, 638–654 (2009).
